# Pre-prescription review system improves prescription safety, efficiency, and cost-effectiveness in pediatric outpatients: a western China study

**DOI:** 10.3389/fphar.2025.1576442

**Published:** 2025-07-24

**Authors:** Bin Yang, Junyan He, Qianbo Li, Xiuling Wang, Lin Song

**Affiliations:** ^1^ Department of Pharmacy, Children’s Hospital of Chongqing Medical University, National Clinical Research Center for Child Health and Disorders, Ministry of Education Key Laboratory of Child Development and Disorders, Chongqing Key Laboratory of Pediatric Metabolism and Inflammatory Diseases, Chongqing, China; ^2^ Department of Information, Children’s Hospital of Chongqing Medical University, National Clinical Research Center for Child Health and Disorders, Ministry of Education Key Laboratory of Child Development and Disorders, Chongqing Key Laboratory of Pediatric Metabolism and Inflammatory Diseases, Chongqing, China

**Keywords:** pre-prescription review system, time efficiency, economic, pharmacist, pediatric

## Abstract

**Objective:**

The study’s objective is to investigate the prescription rationality rate, time efficiency, and estimated economic benefits of a pre-prescription review system (PPRS) in pediatric outpatients in a 5-year retrospective study in western China.

**Method:**

This retrospective before-after study compared data from two phases: pre-intervention phase (2019: PPRS not yet implemented), and post-intervention phase (2020–2023: PPRS fully operational). The study evaluated three key endpoints: primary endpoint was the prescription rationality rate; the secondary endpoints were time efficiency (interval from prescription to drug dispensing) and suggested economic benefits (estimated benefit-cost ratio). Descriptive analyses were conducted to investigate the prescription rationality rate before and after PPRS implementation. The time interval from prescription to drug dispensing was analyzed from 2019 to 2023 using one-way ANOVA. Suggested economic benefit was evaluated under the healthcare system perspective with estimated benefit-cost ratio.

**Results:**

The PPRS implementation was correlated with an increase in the rational prescription rate in outpatient department from 91.19% in 2019 to 98.79% in 2023 and a reduction in time interval from prescription to dispensing from 19.59 min in 2019 to 16.33 min in 2020 (*p* < 0.0001), with sustained lower levels from 2020 through 2023 (16.07 min–16.33 min). Conducting the PPRS was also associated with indicated cost savings, with an estimated benefit-to-cost ratio exceeding 1.64.

**Conclusion:**

The implementation of PPRS was associated with enhanced prescription rationality and time efficiency in pediatric outpatient settings while indicating potential economic benefits. This PPRS is worthy of popularization but needs to be validated across diverse populations and institutions.

## 1 Introduction

Adverse drug events (ADEs) are unintended, harmful occurrences linked to medications, which impact patients’ safety and the quality of healthcare (World Health Organization, 2008). The medication use process encompasses the activities of prescribing, dispensing, administering, and monitoring, which necessitate the collaboration of various healthcare professionals and stakeholders across diverse geographical settings (Manias et al., 2021). If an error occurs at any one stage, harm may occur.

Due to factors such as differences in age and weight, increased inter-patient variability, rapid fluctuations in drug pharmacokinetics, and the frequent use of “off-label” drug indications, pediatric patients exhibit a higher vulnerability to severe adverse effects and mortality compared to adults (Leitzen et al., 2021; Yackey and Stanley, 2019). On the other hand, polypharmacy has become a critical driver of ADE in pediatric healthcare, significantly heightening the risks of drug-drug interactions, dosing miscalculations, and contraindicated combinations (Mengato et al., 2025; Zanin et al., 2024; Fraser et al., 2022). This trend is exacerbated by disease complexity and fragmented care across specialties, leading to uncoordinated prescribing. Such polypharmacy-related errors necessitate robust systemic safeguards like computerized prescription review.

Prescription error is one of the most common and preventable causes of iatrogenic injuries (Parthasarathi et al., 2021; Velo and Minuz, 2009). Pharmacists engage in all phases of the medication process to reduce hospital stays, intensive care requirements, procedure costs, and frequency of preventable ADEs (English et al., 2020; Dawoud et al., 2019). Therefore, examining prescriptions by pharmacists is crucial in detecting errors and averting ADEs in patients. In June 2018, China established new guidelines for prescription review in medical institutions that not only outlined the key principles of prescription review but also emphasized the crucial involvement of pharmacists in the review process (National Health Commission of the People’s Republic of China, 2018). As per the guidelines, medical institutions were encouraged to establish informatized intelligent prescription review systems during the physician ordering process with pharmacist oversight before finalization (National Health Commission of the People’s Republic of China, 2018). And this process occurs before prescriptions are submitted for payment or dispensing.

The PPRS, developed and maintained by referring to pertinent handbooks, guidelines, online databases, and published studies, is a computerized system embedded in our hospital’s Hospital Information System (HIS), aiming to support prescription decisions intelligently. Hence, the PPRS can automatically suggest usage details to doctors when prescribing, pre-screen, and prevent incorrect prescriptions based on various security levels. While certain scholars have focused on the impact of PPRS on medication errors in pediatric inpatient and outpatient settings (Wang et al., 2022; Tan et al., 2022), they have not explored its effects on the time-saving benefits and estimated economic value. The PPRS is still in its early stages, indicating that further investigation is warranted.

This retrospective before-after observational study aimed to investigate whether the implementation of PPRS was associated with improved rational prescription rate, decreased time interval from prescription to dispensing, and resulted in potential economic benefits for the healthcare system.

## 2 Methods

### 2.1 Design and setting

A retrospective observational study was performed at the Children’s Hospital of Chongqing Medical University, a 2480-bed university hospital in western China. The study was divided into two stages: the preparation for pre-prescription review began at the end of 2019 but was not implemented, while the official implementation of pre-prescription review in outpatients started in 2020. The PPRS is staffed by four full-time pharmacists-in-charge, who work 7 days a week from 8:00 a.m. to 5:30 p.m. The pharmacist’s primary responsibilities include conducting real-time pre-prescription reviews when doctors prescribe medications, consulting relevant handbooks, guidelines, online databases, and published studies, maintaining and updating the drug database and prescribing protocols, and collaborating with clinical physicians through communication and training.

### 2.2 Flowchart of PPRS

**FIGURE 1:**
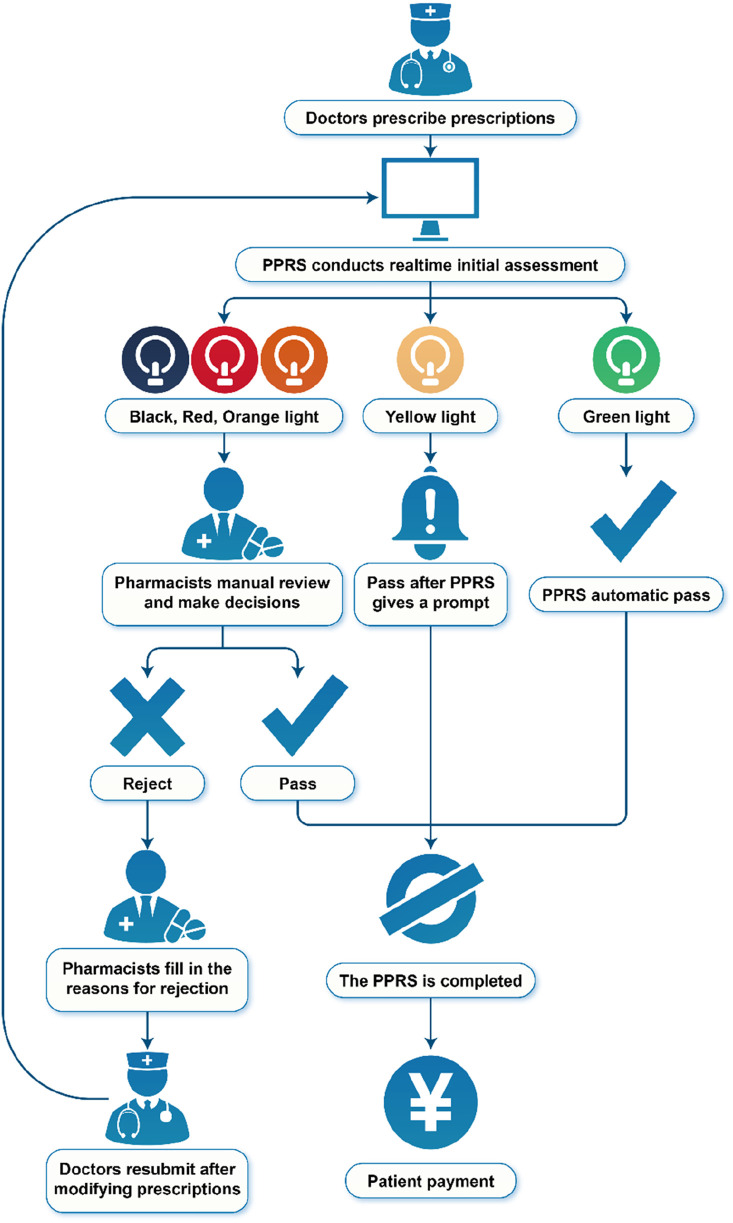
Flowchart of pre-prescription review system.

The PPRS, developed by Sichuan Meikang Medical Software Research and Development Co., Ltd., in Sichuan, China, was an automated system integrated into the HIS, with the goal of providing intelligent prescription decisions. The flowchart of PPRS is presented in Figure 1.

PPRS intervention occurs at the point of prescription entry by physicians. The PPRS automatically conducts a real-time initial assessment during the physician ordering process, with pharmacist oversight prior to finalization. Depending on the severity of the potential outcomes resulting from prescription mistakes, the warning levels were categorized into black, red, orange, yellow, and green. The information regarding commonly associated pharmaceuticals for each warning level can be found in Table 1. Prescriptions deemed appropriate are marked in green and approved for printing and payment. Conversely, if the system detects a potentially inappropriate prescription based on the corresponding warning levels, two distinct scenarios may arise. For prescriptions flagged with a yellow warning, the request will proceed, but details regarding the medication will be communicated to the physicians to alert them to the potential safety issue, allowing for careful review of the prescription. In the case of black, red, or orange warnings, physicians must provide justifications that require approval from pharmacists before the prescription can be processed by the system. The revised prescription will also undergo review. Pharmacists retained full authority to override system alerts based on clinical judgment. All overrides were logged for HIS and PPRS.

**TABLE 1:** Examples of black/red/orange/yellow warnings.

Light	Content	Examples
Black light	a: Strictly prohibited administration frequency/routes of administration	When used to treat juvenile idiopathic arthritis, methotrexate is administered once daily
	b: Age contraindications	Sulfadiazine tablets are indicated for use in infants younger than 2 months
Red light	a: Repetitive use of similar medications	The simultaneous administration of ibuprofen and acetaminophen is employed for the management of upper respiratory tract infections
	b: Prohibited drug interactions	Simultaneous administration of cefoperazone and oral drops of Oulongma (with the excipient ethanol content at 19%)
	c: Allergic reactions to the drug or excipients, or positive skin test results	Prescribe *Saccharomyces* boulardii powder (excipients contain lactose) for patients with lactose intolerance
	d: The eighteen contra-indications and nineteen cautions of traditional Chinese medicine	The prescription of traditional Chinese medicine includes both ginseng and veratrum
	e: Inappropriate concentration of injectable formulations	Potassium chloride injection prepared at >3% (w/v) concentration
	f: Incorrect administration route	The lyophilized thrombin powder is intended for injectable administration
Orange light	a: Surpassing the upper threshold of the prescribed dosage without attaining the maximum therapeutic limit	The cefixime granules were administered three times daily
	b: Falling short of the lower threshold of the recommended dosage without dipping below the minimum effective dose	The cefaclor granules were administered once daily
	c: Employing medications for unapproved indications	Mepolizumab is used for upper respiratory tract infections
Yellow light	a: Certain precautions regarding pharmaceuticals	The compound vitamin B_12_ solution is intended for use in children aged 3 years, but the PPRS alerts the physician that the patient may be too young to perform gargling

### 2.3 Clinical prescription safety

In this study, clinical prescription safety is divided into prescription rationality rate and the quantity of black/red/orange/yellow warnings. The prescription rationality rate reflects the improvement in prescription quality, while the quantity of physician-modified black/red/orange/yellow warnings categorized by risk severity quantifies the efficacy of PPRS in intercepting safety-critical prescription risks that could lead to preventable medication-related harm.

#### 2.3.1 Prescription rationality rate

Prescription evaluation follows the guidelines of the “Regulations on the Management of Hospital Prescription Evaluation (Trial)” (National Health Commission of the People’s Republic of China, 2010). We evaluated the prescription rationality rate by categorizing prescriptions into four types: total prescriptions--all outpatient prescriptions during the study period; essential medications--prescriptions containing ≥1 drug listed in the National Essential Medicines List (2018 Edition) (National Health Commission of the People’s Republic of China, 2018a); antibacterial drugs--prescriptions containing systemic antibacterial agents, excluding topical formulations; traditional Chinese medicine--prescriptions containing ≥1 Chinese patent medicine. This categorization is crucial as the significance and risks associated with different types of medications vary in clinical use. Essential medications serve as the foundation for patient treatment, while the rational use of antibacterial drugs is directly linked to the development of antimicrobial resistance. Additionally, traditional Chinese medicine holds a significant role in pediatric care. Classification was automated using the MeiKang Clinical Pharmacy Management System based on the hospital’s drug master database in the HIS. Rationality criteria were uniformly applied across categories. Rationality rates were calculated as: Rationality Rate = Number of Rational Prescriptions in Category/Total Prescriptions in Category × 100%.

#### 2.3.2 The quantity of black/red/orange/yellow warnings

The MeiKang Clinical Pharmacy Management System automatically saves and extracts all potential issues marked as black/red/orange/yellow warnings in prescriptions from 2020 to 2023, and records the modifications made by physicians to these issues.

### 2.4 Time interval from prescription to drug dispensing

Time interval from prescription to drug dispensing refers to the time for patients receiving prescriptions from doctors and dispensing the medication at the pharmacy, which was automatically retrieved from the HIS of our hospital. After the patient gets the prescription, they can choose among three payment methods: queueing at the hospital’s service window to pay in cash, by bank card, or via WeChat; making payments at the hospital’s self-service machines; or conducting online payments through the hospital’s internet portal. Once the patient pays, the HIS automatically assigns a dispensing window and shows the patient’s name. The medication information is also sent to the patient’s mobile phone. Patients then queue at the pharmacist’s window using the displayed name or the mobile phone reminder.

### 2.5 Estimated cost–benefit analysis

Estimated cost-benefit analysis was performed from a hospital perspective to estimate the indicated savings of the PPRS. The estimated cost-benefit analysis is calculated by dividing the total indicated savings by the total indicated costs. Potential economic costs and indicated savings were calculated in Chinese Yuan (*RMB*).

#### 2.5.1 Indicated savings

In reference to the work of Al-Qudah et al., we improved the assessment method for outpatient ADE costs. Al-Qudah et al. measured the savings of ADEs by summing the average expenses of internal medicine admissions and emergency visits (Al-Qudah et al., 2020). We further postulate that critical issues indicated by black and red alerts, which are not intercepted by pharmacists, may lead to hospitalization; therefore, we will apply average hospitalization costs for these ADEs. Conversely, issues flagged as orange represent less severe concerns that typically only require a general outpatient visit; hence, we will use the average outpatient cost for these instances. The yellow alert serves merely as a reminder for physicians and is not considered in this study.

In the absence of PPRS, when outpatient doctors issued unreasonable prescriptions, it is assumed that pharmacists intercept 99.78% of these prescriptions (Snoswell, 2020; Ness et al., 1994). Consequently, 2.2‰ of the remaining unreasonable prescriptions may result in ADEs. When the PPRS is implemented, the total indicated savings amount to 2.2‰ of the total number of black, red, and orange light issues modified by doctors through the PPRS, multiplied by the cost associated with each ADE.

The formula for calculating the total indicated savings of outpatient ADEs is as follows: Total indicated savings = (N_b_ + N_r_) × 2.2‰ × C_h_ + N_o_ × 2.2‰ × C_o_. Where: N_b_ = Number of black light, N_r_ = Number of red light, N_o_ = Number of orange light, C_h_ = Average hospitalization cost for the year, C_o_ = Average outpatient cost for the year.

According to the statistical data released in the Health Development Statistical Bulletin of China for the years 2020–2023, the average outpatient and hospitalization costs were 324.4 yuan and 10,619.2 yuan, respectively, in 2020 (National Health Commission of the People’s Republic of China, 2021). In 2021, these figures increased to 329.2 yuan and 11,002.9 yuan (China Government Website, 2022). By 2022, the averages rose to 342.7 yuan and 10,860.6 yuan (National Health Commission of the People’s Republic of China, 2023). And in 2023, they reached 361.6 yuan and 10,315.8 yuan (National Health Commission of the People’s Republic of China, 2024).

#### 2.5.2 Indicated costs

Indicated costs = Pharmacists (resource expenses: salaries) + Hardware (four computers) + PPRS (purchase and maintenance).

According to the “2021 China Hospital Salary Survey Report” published by Ding Xiang Yuan Community (2021), pharmacists’ earnings are determined by the mean pay of Chinese hospital staff in 2020, which stands at 197,422 RMB. This figure represents the yearly income for pharmacists in 2020. Following this, revisions are implemented on pharmacists’ salaries for 2021, 2022, and 2023 in line with China’s Gross Domestic Product growth.

This study merely conducted a basic cost-benefit ratio analysis. Savings were limited to direct medical fees (outpatient and inpatient fees), without considering indirect fees (e.g., productivity loss, transportation) or conducting a probability sensitivity analysis. The ADE incidence rate was derived from literature (Ness et al., 1994; Snoswell, 2020) and might not fully reflect the actual situation. These estimates should be regarded as indicators of potential savings rather than a comprehensive pharmacoeconomic evaluation.

### 2.6 Data collection

The time from prescription to dispensing was obtained automatically from the HIS of our hospital. The MeiKang Clinical Pharmacy Management System automatically extracted the outpatient prescription rationality rates and the quantity of black, red, yellow, and orange lights modified by the physicians.

### 2.7 Statistical analysis

Descriptive statistical analysis was conducted on the prescription rationality rate and the number of modifications made by physicians regarding the black/red/orange/yellow warnings. One-way ANOVA was employed to compare the changes in the time from prescription to dispensing between 2019 and 2023. All statistical analyses were conducted with EmpowerStats (www.empowerstats.com, X&Y solutions, Inc. Boston MA). The significance level was set at p < 0.05.

## 3 Results

### 3.1 Prescription safety outcomes

#### 3.1.1 Rationality rate improvement

**FIGURE 2:**
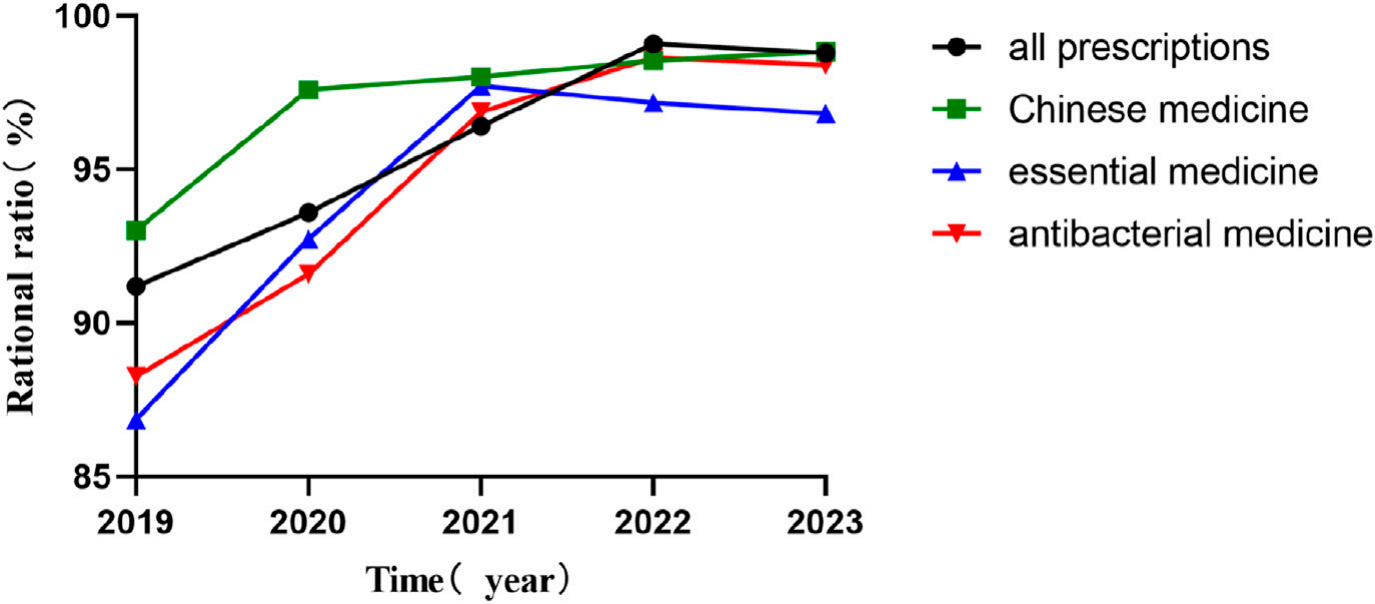
Rationality rates of prescription categories before (2019) and after (2020–2023) PPRS implementation.

Figure 2 compares the rationality rates of prescription categories before (2019) and after (2020–2023) PPRS implementation. The rationality of the four types of prescriptions all improved. From 2019 to 2023, the rationality of total prescriptions increased from 91.19% to 98.79%; traditional Chinese medicine prescriptions rose from 93.02% to 98.83%; essential medicine prescriptions went up from 86.86% to 96.82%; and antibacterial medicine prescriptions improved from 88.25% to 98.40%.

#### 3.1.2 Alert intervention

As shown in Table 2, the total volume of PPRS-intercepted alerts increased progressively from 99,684 in 2020 to 274,060 in 2023. Red and orange warnings constituted the primary categories, accounting for 44.20%–55.81% and 42.82%–52.83% of annual totals, respectively. Black light errors demonstrated consistent annual growth in both absolute frequency (1,374 to 11,222 cases) and proportional representation (1.38%–4.09%).

**TABLE 2:** PPRS alert volume and proportion by severity level (2020–2023).

Year	Black light		Red light		Orange light		Yellow light		Total	
	N	%	N	%	N	%	N	%	N	%
2020	1,374	1.38	55,630	55.81	42,680	42.82	0	0.00	99,684	100
2021	6,596	2.96	98,383	44.20	117,598	52.83	0	0.00	222,577	100
2022	9,207	4.03	103,347	45.20	112,901	49.37	3,214	1.41	228,669	100
2023	11,222	4.09	131,324	47.92	129,582	47.28	1932	0.70	274,060	100

### 3.2 Time efficiency gains

**FIGURE 3:**
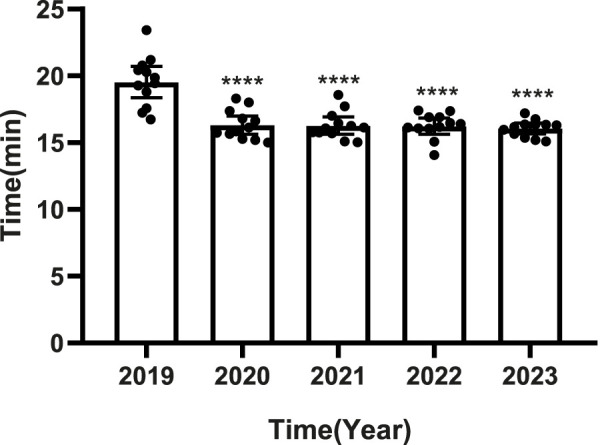
Time interval from prescription to dispensing before (2019) and after (2020–2023) PPRS implementation. •: Monthly data; □: Annual mean; ****p < 0.0001 vs. 2019.

PPRS intervention was associated with reducing the time for patients from prescription to dispensing (Figure 3). The most substantial decrease occurred between 2019 (pre-intervention: 19.59 min) and 2020 (first intervention year: 16.33 min) (*p* < 0.0001). From 2020 to 2023, the time remained consistently lower (range: 16.07 min–16.33 min) with no significant inter-annual differences (*p* > 0.05).

### 3.3 Estimated cost–benefit analysis

The estimated cost–benefit analysis of PPRS showed a yearly rise, climbing from 1.62 in 2020 to 3.41 in 2023, with a cumulative increase of 110% (Table 3). Over the same period, the indicated savings rose from 1.36 million yuan to 3.34 million yuan, while the indicated costs went up from 0.83 million yuan in 2020 to 0.97 million yuan in 2023. In terms of indicated cost breakdown, hardware and system maintenance costs stayed constant. As for pharmacist costs, they experienced a moderate annual rise due to salary adjustments.

**TABLE 3:** Estimated operational costs and indicated savings of PPRS from 2020 to 2023.

Years	Indicated costs (×10^4^ RMB)			Indicated savings (×10^4^ RMB)	Estimated benefit-to-cost ratio
	Pharmacists	Hardware	PPRS		
2020	78.97	0.12	4	136.22	1.62
2021	85.64	0.12	4	262.63	2.89
2022	88.21	0.12	4	277.44	2.97
2023	92.80	0.12	4	333.81	3.41

## 4 Discussion

This study, in a 5-year retrospective before-after study analysis, is the first to evaluate the impact of PPRS on prescription rationality rate, time efficiency, and potential economic benefits in pediatric outpatient settings in western China. It revealed that PPRS was closely linked to a significant rise in prescription rationality, with rates improving from 91.19% in 2019 to 98.79% in 2023. Furthermore, the average time for patients from prescription to dispensing decreased from 19.59 min in 2019 to 16.33 min in 2020 (*p* < 0.0001). PPRS also had potential economic value with estimated benefit-to-cost ratio rising yearly (from 1.62 in 2020 to 3.41 in 2023). In summary, the implementation of PPRS shows multidimensional value in improving pediatric medication use in western China. PPRS is worthy of popularization but needs to be validated across diverse populations and institutions.

Similar to research conducted by other scholars, PPRS contributes to rationalizing outpatient prescriptions (Zhang et al., 2024). However, findings from our study reveal that the enhancement in prescription rationality did not exhibit a steady increase over the years but rather displayed gradual fluctuations. The fluctuations may be attributed to several factors. First, the knowledge base may lag in updates, preventing the timely incorporation of the latest drug information and treatment guidelines. This delay can lead the system to misclassify some new rational drug uses as unreasonable, resulting in “false positives” and consequently affecting the accurate assessment of prescription rationality (Ma et al., 2021). Second, overly strict or imprecise review rules within the system may inadvertently block prescriptions that are, in fact, reasonable, while failing to effectively identify potentially unreasonable ones. Third, the long-standing medication habits and experiences of some physicians may pose challenges in adjusting to the new prescription guidelines and rational drug use requirements within a short timeframe (Liao et al., 2019). Lastly, some physicians may exhibit an overreliance on feedback from the PPRS, resulting in superficial modifications to flagged prescriptions without conducting a thorough analysis of the underlying reasons, thereby lacking a comprehensive understanding of the principles and requirements of rational drug use.

Notably, there is a lack of prior research addressing the time from prescription receipt to dispensing. This research introduces the time interval for the first time. Our research indicates a reduction in time interval from prescription to dispensing from 19.59 min in 2019 to 16.33 min in 2020. The reduction can be primarily attributed to prompt adjustments made by doctors to rectify any inappropriate prescriptions flagged by the PPRS, thereby preventing patients from experiencing the inconvenience of multiple visits between the pharmacy and the clinic due to irrational prescriptions. The Chinese government actively supports the growth of online payment systems in public hospitals (China Government Website, 2018). As a result, patients now have options beyond traditional counter payments. They can utilize self-service machines or online payment platforms like Alipay and WeChat Pay. This shift drastically reduces waiting times for consultations. However, this study overlooks how these new payment methods affect consultation durations. In addition, the impact of different departments and varying degrees of illness complexity on consultation time was not considered. Future studies will conduct a more in-depth examination of the precise influence of PPRS on the duration of patient consultations, while controlling for the potential confounding effects of payment variables as well as the effects of different departments and the complexity of illnesses. From 2020 to 2023, the time reduction remained consistently lower (range: 16.07 min–16.33 min) with no significant inter-annual differences. It indicated that in the early stages of operation, the PPRS can quickly and steadily enhance the process. However, its optimization of reduction time from prescription to dispensing isn't a continuous process. The lack of further significant reduction after 2020 may indicate that the system reached its maximal efficiency impact within the existing workflow framework, or that other bottlenecks (e.g., payment processing, pharmacy staffing) became limiting factors.

To our understanding, this study is the initial one to show that PPRS has a positive potential economic impact on drug safety concerning the avoidance of ADEs in pediatric outpatients in western China. The estimated cost-effective outcomes observed in our study closely resemble those reported following pharmacist interventions. Zhiwei Bao et al., show that the cost-benefit ratio of pharmacist interventions on sampled outpatient prescriptions in a Chinese teaching hospital was always more than 1.14 (Bao et al., 2018). Sarah Wilkes et al., report that conducting medication reviews showed a positive cost–benefit ratio of 9.7 (Wilkes et al., 2022). However, it is important to note that another study reported no significant reduction in medical expenditures associated with ADEs following the introduction of the PPRS (Kim et al., 2018). This result may be partially attributed to the study’s narrow focus on NSAIDs-related ADEs, such as gastrointestinal ulcers and bleeding, without considering other types of ADEs (Kim et al., 2018). In this research, while our estimated benefit-to-cost ratios (1.62–3.41) suggest that PPRS may be economically favorable, these findings require cautious interpretation. The analysis serves as an initial indication of cost-saving potential but lacks elements of full economic assessments, such as sensitivity analyses or inclusion of societal costs. Future studies incorporating longitudinal ADE tracking and multi-faceted cost accounting would strengthen the economic evidence.

While our analysis suggests PPRS was associated with enhanced prescription rationality and time efficiency while indicating potential economic benefits, its implementation reveals critical limitations in replacing human expertise. On the technical front, machines can aid in boosting efficiency but cannot fully substitute pharmacists. The reasons are as follows: First, the system depends on pharmacists for rule library maintenance. Second, in complex cases, pharmacists need to make judgments based on their clinical experience, such as evaluating drug interactions during polypharmacy. Last, regarding humanistic care, machines can’t replace the empathy and trust between doctors, pharmacists, and patients in communication. Pharmacists play an irreplaceable role in interpreting the intent of reviews, individualized treatment, and humanistic care (Martini et al., 2024). From an ethical perspective, the system is merely a decision-support tool, with the ultimate responsibility for prescribing still lying with physicians and pharmacists (World Health Organization, 2024). The future model should be one of “human-machine collaboration” to jointly promote the safe medication use in children (Martini et al., 2024).

This study has several limitations. First, it’s a single-center design at a tertiary comprehensive children’s hospital located in western China, which may introduce selection bias and limit the ability to generalize findings to other healthcare institutions or populations. Second, the system recorded aggregate counts of black/red/orange/yellow alerts, but the specific distribution of error subtypes was not systematically extracted in our dataset. Through analyzing the specific error types corresponding to different color alerts, we can identify priorities for clinical safety optimization, verify system rules, and allocate resources. Future studies should incorporate granular error-type analysis to identify priority areas for clinical education. Third, the study merely conducted a basic cost-benefit ratio analysis rather than a comprehensive pharmacoeconomic evaluation.

In summary, PPRS implementation in pediatric outpatient clinics in western China was associated with enhanced prescription rationality and time efficiency while indicating potential economic benefits. These findings establish PPRS as a vital tool for optimizing medication safety and healthcare efficiency. This study provides empirical evidence for scaling such intelligent systems in resource-limited settings and contributes a replicable framework to global digital health practices, though validation across diverse populations and institutions remains warranted.

## Data Availability

The raw data supporting the conclusions of this article will be made available by the authors, without undue reservation.
